# How should CT coronary angiography be integrated into the management of patients with chest pain and how does this affect outcomes?

**DOI:** 10.1093/ehjqcco/qcv027

**Published:** 2015-11-12

**Authors:** Mhairi K. Doris, David E. Newby

**Affiliations:** Centre for Cardiovascular Science, University of Edinburgh, 49 Little France Crescent, Edinburgh EH16 4SB, UK

**Keywords:** Computed Tomography Coronary Angiography, Outcomes, Chest Pain, Coronary Heart Disease

## Abstract

When examining the role of a diagnostic test in clinical practice, consideration must be placed not only on the accuracy of the result, but also its impact on patient care and outcomes. Proving a direct effect on outcomes may be difficult because the impact of the diagnostic test largely depends on the clinician's interpretation and consequent actions as well as the patient's response to changes in their diagnosis, investigations, and treatment. Recent major clinical trials of symptomatic patients with suspected coronary heart disease (CHD) have shown that computed tomography coronary angiography (CTCA) can markedly clarify the diagnosis and lead to major changes in patient investigation and management including the use of invasive angiography, preventative therapies, and coronary revascularization. Thus, when added to our existing clinical tools, such as exercise electrocardiography, CTCA represents a powerful method of identifying and excluding CHD. Furthermore, it can identify patients with prognostically relevant non-obstructive CHD and, with recent technological advances, will be able to assess the functional impact of anatomically detected coronary artery stenoses. Overall, the routine integration of CTCA into the investigation of patients with chest pain improves clinical diagnostic certainty that has led to better targeting of investigations and evidence-based treatments that have ultimately translated into improved clinical outcomes.

## Introduction

When introducing a diagnostic test, the first step is to establish its accuracy in comparison with the gold-standard referent investigation. Historically, this has often been the main prerequisite for its adoption into clinical practice. However, increasingly diagnostic nvestigations are required to demonstrate not only diagnostic accuracy but also the clinical and cost effectiveness of the findings on subsequent patient diagnosis, risk stratification, investigations, treatments, and finally clinical outcomes. It is these serial downstream effects on clinical management and outcomes that ultimately define the clinical utility of a diagnostic test.

The rapid technological advances of computed tomography coronary angiography (CTCA) have raised promise that this imaging modality may fulfil the role of a gold-standard non-invasive investigation of chest pain. Recent research has focused on investigating the merit of integrating CTCA into patient management by aiming to determine its effect on treatment and clinical outcomes.

## Initial assessment and management

Chest pain is a common and often concerning symptom that frequently precipitates attendance to a primary care physician with onward referral to specialist cardiology services. The aim of referral is to ascertain the cause of symptoms and to identify those patients with angina pectoris secondary to coronary heart disease (CHD). This would in turn lead to risk stratification and the initiation of evidence-based treatments, with the ultimate goal of improving symptoms and reducing the risk of future adverse cardiovascular events.

Whilst cardiology clinics are effective at identifying high-risk patients with angina, a significant number of patients can be misdiagnosed. Specifically, one-third of CHD events occur in patients who have been initially diagnosed as having ‘non-cardiac’ chest pain.^1^ These patients are younger and less likely to have typical symptoms. Furthermore, a report investigating outcomes of patients attending cardiology clinics with new onset chest pain found that, when applying national guideline recommendations, two-thirds of patients were excluded from further cardiac investigation due to the perception of low risk. However, 10% of patients not offered investigation were subsequently diagnosed as having significant CHD.^2^ This highlights the fact that misclassification can lead to adverse outcomes and reflects the need for a clearer diagnosis in low-risk populations. Indeed, this represents the majority of patients attending cardiology clinics with recent onset chest pain and a test that could reliably exclude CHD in this group of patients may not only provide reassurance but also reduce adverse outcomes.^1,2^

An initial assessment of the patient with chest pain often involves the estimation of cardiovascular risk using traditional risk estimation models, providing the clinician with a guide on which to base the choice of diagnostic pathway. The Diamond and Forrester prediction rule was first published in 1979 and continues to form the basis of current international guidelines.^3,4^ However, such traditional risk factor models overestimate the probability of CHD in the general population and especially in women.^3,5,6^ This can lead to the over-investigation of patients or the initiation and maintenance of unnecessary medical treatments.

Following meticulous history taking and an estimation of probability, many clinicians will seek the support of a diagnostic test in order to confirm or to exclude the diagnosis of angina pectoris secondary to CHD. This approach is supported by international guidelines.^7,8^

## Current guidelines

There exists an abundance of non-invasive testing strategies that serve to further improve risk stratification and refine the probability of myocardial ischaemia secondary to CHD. At present, there is no widely adopted strategy of a single gold-standard non-invasive investigation. Indeed, the performance of an individual investigation in the clinical setting is closely dependent on the pretest probability of CHD. In selecting a test, a clinician must use this information in order to select the most appropriate investigation to maximize diagnostic certainty and to minimize the risk of false-positive or false-negative results.

Evidence has demonstrated that selective referral for angiography based on the results of non-invasive testing is both safe and cost effective.^9,10^ The National Institute for Clinical Excellence (United Kingdom) guidelines recommend invasive angiography for diagnostic purposes in patients with a pretest likelihood of CHD of 61–90%.^11^ The European and American guidelines reserve invasive angiography for those patients with severe symptoms despite medical therapy, left ventricular dysfunction, or suspected high-risk disease.^7,8^ Whilst guidelines across the UK, Europe, and the USA differ in their recommended diagnostic pathway, a common recommendation is the utilization of a functional testing strategy. However, the guidelines are inconsistent and recommend different approaches (*Table 1*). Moreover, prevalent practices are at odds with these guidelines. For example, the American College of Cardiology/American Heart Association guidelines primarily recommend exercise electrocardiography, whilst the majority of North American clinicians will undertake nuclear perfusion scans as the non-invasive stress test of choice.^12^ Conversely, the European Society of Cardiology guidelines suggest a ‘preference’ for stress imaging tests above exercise electrocardiography where expertise and resources are available. These recommendations have been made based on empirical clinical practice and studies assessing comparative diagnostic accuracy and patient risk stratification but not on clinical outcomes.

**Table 1:** Current guideline recommendations

Guideline	Risk prediction model	Estimated likelihood	Recommendation for further investigation	Recommendation for CTCA
European Society of Cardiology^7^	Diamond–Forrester Model (updated and extended to include 70 years and older)	<15%	Can be managed without further testing	Alternative to stress imaging for ruling out CHD in patients in whom good image quality can be expected
		15–65%	Exercise ECG if feasible. Stress imaging preferable	
		66–85%	Non-invasive functional test	
		>85%	OMT and risk stratification	
National Institute for Clinical Excellence (NICE) United Kingdom^11^	Diamond–Forrester Model Duke Database	<10%	Consider other causes	
		10–29%	CT calcium scoring	If calcium score 1–400
		30–60%	Functional Imaging	
		61–90%	Invasive angiography	
		>90%	Manage as angina	
American Heart Association/American College of Cardiology^8^	Diamond–Forrester Model Coronary Artery Surgery Study Duke Database Recommendation based on ability to exercise, resting ECG, and history of previous revascularization	Low to intermediate	If resting ECG interpretable and able to exercise—exercise ECG. if unable to exercise—Pharm stress ECHO	Incapable of moderate physical activity or have disabling comorbidity
		Intermediate	Exercise ECG. If unable to exercise—Pharm stress MPI/ECHO or Pharm CMR or CCTA	May be reasonable for patients who have at least moderate physical functioning/no disabling comorbidity
		Intermediate to high	If able to exercise—MPI or ECHO with exercise or pharm CMR. If unable to exercise—Pharm stress MPI/ECHO or Pharm CMR or CCTA	If stress testing contra-indicated or unable to exercise

OMT, optimal medical therapy; MPI, myocardial perfusion imaging; CMR, cardiac magnetic resonance.

## Functional testing

### Diagnostic accuracy

In a registry of over 600 000 patients undergoing angiography, results of non-invasive testing had only a weak correlation with likelihood of obstructive disease, and patients with a positive result of a non-invasive test were only moderately more likely to have obstructive CHD compared with those who did not undergo any testing.^13^ In this patient population, the most utilized non-invasive test was single photon emission computed tomography (SPECT) myocardial perfusion imaging (performed in 78.1%), whereas CTCA was performed in a minority (2.1%). Younger patients, women, and those with atypical symptoms were more likely to have non-obstructive coronary artery disease.^13^

A recent meta-analysis assessed the diagnostic accuracy of myocardial perfusion imaging compared with invasive angiography plus fractional flow reserve (FFR) and found that the sensitivity and specificity of myocardial perfusion imaging with SPECT in detecting obstructive disease were 74 and 79%, respectively, whereas stress echocardiography (ECHO) yielded a sensitivity and specificity of 69 and 84%, respectively.^14^ In this study, stress myocardial perfusion with magnetic resonance imaging, CT, and positron emission tomography (PET) performed better, with substantially higher diagnostic accuracy (*Table 2*). Indeed, in head-to-head comparisons, magnetic resonance has outperformed SPECT with a negative predictive value of 91% compared with 79%, respectively.^18^

**Table 2:** Diagnostic accuracy of functional tests

First author/year	Study design	Aims	Patients (*n*)	Main findings
Mahajan *et al.*, 2010^15^	Meta-analysis	To compare diagnostic accuracy of MPI and SE for the diagnosis of left main stem and triple vessel disease	3713	SE had higher pooled sensitivity than MPI (94 vs. 75%, *P* < 0.001). No difference in pooled specificity for SE and MPI (40 and 48%, *P* = 0.16)
Chinnaiyan *et al.*, 2012^16^	Prospective Non-randomized registry data	To assess correlation and compare the diagnostic performance of CTCA and stress testing in patients undergoing ICA	6198	Stress test results did not accurately predict CHD on ICA. Only 59% of patients with abnormal stress tests had >50% stenosis on ICA^a^
Patel *et al.*, 2014^13^	Observational Registry Data	To investigate relationship between clinical characteristics, NIT results, and likelihood of CHD	661 063	NIT findings had minimal incremental value beyond clinical factors for predicting obstructive disease (*C-index* = 0.75 *for clinical factors vs.* 0.74 *for NIT findings*)
Neglia *et al.*, 2015^17^	Prospective multicentre, non-randomized	To compare the diagnostic accuracy of functional imaging and CTCA in detecting significant CHD defined by ICA	475	MPI sensitivity and specificity 74 and 73%, respectively.^a^ Stress ECHO/CMR sensitivity and specificity 49 and 92%, respectively
Takx *et al.*, 2015^14^	Meta-analysis	Comparison of non-invasive imaging (functional and CTCA) with ICA and FFR in detection of functionally significant CHD	2048	MRI sensitivity and specificity 89 and 87%, respectively. PET 84 and 87% CT 88 and 80% SPECT 74 and 79% ECHO 69 and 84%
Greenwood *et al.*, 2012^18^	Prospective cohort study	To investigate the diagnostic accuracy of CMR and compare CMR and SPECT	752	CMR sensitivity 87% and specificity of 83% Sensitivity of SPECT 67% and specificity 83%

NIT, non-invasive tests; ICA, invasive coronary angiography; FFR, fractional flow reserve.

^a^No information regarding location and degree of positive stress tests.

### Risk stratification

Functional testing is a predictor of clinical outcomes. Patients with a low-risk exercise electrocardiogram (ECG) have an annual cardiovascular mortality of <1%.^7^ Some evidence has suggested that myocardial perfusion imaging adds incremental prognostic value over standard diagnostic tests including electrocardiography and ECHO.^19^ Furthermore, a normal perfusion scan is associated with an excellent prognosis, even in the presence of anatomically detected CHD.^20^ In a meta-analysis of the prognostic value of functional testing, the negative predictive value of myocardial perfusion imaging for myocardial infarction (MI) and cardiac death was 98.8% over 3 years of follow-up, with an annualized event rate of 0.45% for a negative test (*Table 3*).^21^

**Table 3:** Functional testing and risk stratification

First author, year	Study design	Aims	Patients (*n*)	Main findings
Metz *et al.*, 2007^21^	Meta-analysis	To determine prognostic value of normal exercise MPI texts and SE	11 029	NPV for MI and cardiac death 98.5% for MPI and 98.4% for SE. Annualized event rates 0.45% (MPI) and 0.54% (SE)
Daly *et al.,* 2006^6^ Euro heart Survey	Prospective observational cohort study	To identify key prognostic features in CHD and construct score to aid risk prediction	3031	Having no stress test associated with increased risk of death or MI (HR 3.78, 95% CI 2.04–7.00). Positive stress test associated with slightly increased risk (HR 1.43, 95% CI 0.76–2.70)
Gimelli *et al.*, 2009^19^	Observational cohort study	To investigate the prognostic value of MPI with gated SPECT	676	Perfusion abnormalities independent predictor of event free survival (SDS HR 1.15, 95% CI 1.03–1.27)
Sicari *et al.*, 2003^22^	Multicentre prospective observational study	To investigate the prognostic value of stress ECHO	7333	Patients with negative SE at low risk of death (<1%/year). Positive test associated with increased risk of cardiac mortality (RR 2.2, 95% CI 1.6–3.1)
Candell-Riera *et al*., 2013^23^	Prospective observational study	To investigate the incremental prognostic value of MPI SPECT compared with exercise electrocardiography	5672	Adding MPI SPECT to exercise ECG improves prediction of major cardiovascular events but does not improve prediction of death
Piccini *et al.,* 2010^24^	Prospective observational study	To investigate whether SPECT MPI enables risk stratification for SCD in patients with CHD and LVEF > 35%	4865	The addition of perfusion data associated with increased discrimination for SCD events (C-index 0.728)

SCD, sudden cardiac death; SE, stress echocardiography; SDS, summed difference score, indicating the extent of reversible perfusion defects; NPV, negative predictive value.

Whilst evidence has concluded that a negative stress test correlates with a favourable prognosis, the relationship between a positive result and adverse outcome is less clear. Results from the Euro Heart Survey demonstrated that, whilst not having any functional assessment was an indicator of increased risk, a positive result from a non-invasive stress test was not associated with an adverse outcome.^6^ The weak correlation between a positive stress test and adverse outcome reflects the finding that stress testing strategies are less reliable in accurately diagnosing CHD.^16^

## Selection of patients for invasive coronary angiography

Despite guidelines recommending the use of non-invasive tests to identify and risk stratify those patients with a high likelihood of CHD, a large proportion of diagnostic angiograms are normal. In a study of 398 978 patients throughout 663 hospitals in America, only 38% of patients undergoing elective angiography had obstructive CHD and 39% had normal coronary arteries.^25^ Similarly, in a multicentre international trial throughout European centres, only 42% of 2260 patients undergoing elective angiography had evidence of obstructive CHD.^5^ This reflects the lack of certainty regarding the diagnosis and the residual concern of missing underlying CHD. There is therefore a major need for an improved diagnostic strategy and improved patient selection for invasive angiography.

## CTCA in the investigation of stable chest pain

The diagnostic accuracy of CTCA has been demonstrated in large multicentre studies that have compared this imaging modality with invasive angiography.^26,27,28^ The results have demonstrated that, in the detection of CHD, CTCA has a sensitivity and specificity which is similar to invasive coronary angiography. However, its positive predictive value in detecting severe stenosis is lower, and the degree of stenosis can be overestimated especially in the presence of marked coronary calcification.

Following the emergence of evidence highlighting the comparable diagnostic accuracy of CTCA when compared with invasive angiography, research has now focused on the clinical application of CTCA and its role in patient management and prognosis. Two large randomized controlled trials have recently addressed this question.

### The PROMISE trial

The PROspective Multicenter Imaging Study for Evaluation of chest pain (PROMISE) trial was a large multicentre study of 10 003 participants undergoing non-invasive investigation for suspected CHD who were randomized to an anatomical testing strategy with CTCA or a functional testing strategy, which included exercise electrocardiography, stress ECHO, or radionuclide perfusion imaging.^12^ The primary endpoint was a composite of all-cause mortality, MI, hospitalization for unstable angina, and major complications of cardiovascular procedures. Secondary endpoints included invasive catheterization showing normal coronary arteries.

The study population had an intermediate risk of CHD with a mean pretest likelihood of obstructive CHD of 53%. Only 12% of the population had typical angina, whereas 11% had non-anginal chest pain. A proportion of patients (27%) had a primary symptom other than chest pain including breathlessness, fatigue, weakness, or palpitations. The choice of functional testing varied, with two-thirds of patients undergoing radionuclide perfusion imaging and 10% undergoing exercise electrocardiography.^12^

The PROMISE trial reported that compared with functional testing, CTCA led to an increase in invasive coronary angiography, although it was less likely to demonstrate normal coronary arteries (4.3 vs. 3.4%; *P* = 0.02) and more likely to lead to coronary revascularization at 90 days (6.2 vs. 3.2%; *P*<0.001).^12^ At 12 months, the risk of death or non-fatal MI was lower in the CTCA group [hazard ratio (HR), 0.66; 95% CI, 0.44–1.00; *P* = 0.049], although this benefit did not persist throughout study follow-up. Ultimately, the event rate in PROMISE was lower than expected (3%) for the pre-specified analysis, and there was no significant difference in outcomes between the two patient groups.^12^

### The SCOT-HEART trial

The Scottish COmputed Tomography of the HEART (SCOT-HEART) trial recruited patients with suspected angina pectoris due to CHD from cardiology clinics and randomized participants (1 : 1) to CTCA plus standard care or standard care alone. This served to investigate the complementary role of CTCA in addition to other clinical tools, as opposed to a direct head-to-head comparison with functional testing strategies.^29^ All participants underwent clinical evaluation including cardiovascular risk assessment. Clinicians were asked to document whether the patient was diagnosed with (i) CHD and (ii) angina pectoris secondary to CHD, as well as their confidence in these diagnoses at both baseline and 6 weeks of follow-up.

The SCOT-HEART trial recruited a broad and representative population of patients referred to the cardiology clinic and included 40% of all patients referred and 47% of those eligible for trial participation. Indeed, this trial specifically included patients who had previously been excluded from diagnostic accuracy studies, such as those with high calcium scores, high body mass index, or atrial fibrillation. Importantly, the majority of patients (85%) underwent exercise electrocardiography in the clinic. This was abnormal in 15% of patients and inconclusive in a further 15%.^29^

At baseline, the attending clinician diagnosed 47% of patients as having CHD and 36% as having angina secondary to CHD. By 6 weeks, the diagnosis changed in 1 of the 4 patients who underwent CTCA compared with only 1% in the standard care group. Specifically, the use of CTCA increased the certainty of the diagnosis of angina secondary to CHD. Interestingly, the overall diagnostic rate of CHD increased, whilst the diagnosis of angina pectoris secondary to CHD appeared to fall with the use of CTCA.

Changes in diagnosis led to alterations in further investigations, with CTCA leading to the cancellation of 121 functional tests and 29 invasive angiograms. Whilst the use of CTCA was associated with an early rise in referrals for invasive angiography (*n* = 94), the majority of these patients had obstructive disease and over half were referred for surgical revascularization due to the presence of high-risk disease.^29^

Consistent with the changing patterns of diagnoses, the use of CTCA was associated with an increase in recommendations for preventive therapy in patients with documented CHD, whilst the use of unnecessary anti-anginal medication fell with the exclusion of obstructive disease. By clarifying and excluding the diagnosis of CHD, unnecessary medications were discontinued, and this may have important implications for patients' health-related quality of life.

Similar to the PROMISE trial, the overall event rate was low in the SCOT-HEART trial, with a 2% overall absolute event rate during 1.7 years of follow-up. This is reflective of the fact that the majority of patients had either normal coronary arteries or non-obstructive CHD, and only 30% were ultimately diagnosed with angina pectoris due to CHD. However, despite this, there was an apparent reduction in CHD death or non-fatal MI with CTCA [38% relative risk (RR) reduction; *P* = 0.0527].^29^

The observed low event rate reflects the generally good prognosis of patients with recent onset stable chest pain and implies a positive effect of current treatment. This also highlights that documenting a clear improvement in prognosis through the effect of a diagnostic test can be challenging. Nevertheless, the results from the SCOT-HEART trial demonstrate that CTCA plays an important role in clarifying the diagnosis of angina pectoris secondary to CHD and leads to important changes in further management, which may ultimately reduce coronary events.

## CTCA in the investigation of unstable chest pain

As well as determining the role of CTCA in the investigation of recent onset stable chest pain, investigators have also sought to determine its value in the Emergency Department setting. The majority of patients presenting to the Emergency Department with acute chest pain do not have an acute coronary syndrome, with pain attributable to non-cardiac causes.^30,31^ However, because of diagnostic uncertainty, many patients are admitted to hospital for a period of monitoring, serial ECGs and biochemical markers, and specialist review. Chest pain accounts for up to 1 in 4 acute hospital admissions,^30^ and population-based rates of hospitalization for suspected cardiovascular disease have been increasing over the past decade.^32^ With such a high negative predictive value, a clear strength of CTCA lies in the reliable exclusion of significant CHD, especially in the low-to-intermediate risk population. This has driven research to investigate the role of early CTCA in the triage and management of low-risk patients with acute chest pain.

The CT-STAT trial compared the use of CTCA with radionuclide myocardial perfusion imaging in the triage of low-risk patients with acute chest pain and demonstrated that the use of CTCA resulted in a 54% reduction in time to diagnosis and 38% reduction in total Emergency Department costs of care. These findings also demonstrated that the presence and severity of atherosclerotic plaque on CTCA were predictive of acute coronary syndrome.^31^

The Rule Out Myocardial Infarction using Computer Assisted Tomography (ROMICAT-II) trial randomly assigned 1000 patients with symptoms suggestive of acute coronary syndrome but negative initial troponin tests and non-ischaemic ECG changes to early CTCA or to standard treatment in the Emergency Department. CTCA led to a reduction in mean length of hospital stay by 7.6 h, and a greater proportion of patients were discharged directly from the Emergency Department (47 vs. 12% *P* < 0.001). This had no adverse effect, and there were no cases of missed diagnosis of acute coronary syndrome.^33^

Patients with a negative CTCA have an excellent prognosis, with low event rates and a ‘warranty period’ that can extend for a number of years.^34,35,36^ Therefore, in addition to facilitating safe and time-efficient discharge, its integration into the management of patients in the acute setting may provide the opportunity to reassure both patients and clinicians of the exclusion of CHD.

## Cost effectiveness

Coronary heart disease represents a significant economical burden to the European Union and the rest of the world. In the European Union, CHD is estimated to cost the economy €60 billion each year, with 33% of this sum attributable to direct healthcare costs. Furthermore, CHD represents an important cause of disability, accounting for 8% of all disability adjusted life years.^37^

Whilst invasive coronary angiography remains the gold standard for the diagnosis of CHD, this is an expensive test associated with a small yet significant risk of major complications.^38^ Economic assessments have concluded that selective referral for invasive coronary angiography is cost effective.^7,8^ However, the proportion of normal invasive angiograms in current practice may suggest poor selection of patients for an initial invasive assessment, or reflect the poor diagnostic accuracy of currently selected non-invasive tests. CTCA, especially when used in in patients with a low-to-intermediate pretest likelihood of CHD, has the potential to improve selection of patients for invasive testing or revascularization, avoiding unnecessary risks and costs associated with invasive angiography. Furthermore, its use in the Emergency Department can allow cost- and time-efficient discharge of patients. Nonetheless, when considering the cost effectiveness of CTCA in routine clinical care, and as a gatekeeper to invasive angiography, further evidence is needed to ascertain fully the resulting healthcare costs, including the impact on downstream testing.

## Ideal diagnostic pathway

From current evidence, no single non-invasive test has achieved the diagnostic accuracy and clinical utility to merit use as a single gold-standard investigation. Whilst evidence has demonstrated that revascularization according to the functional impact of atherosclerosis improves outcomes over assessing the degree of stenosis alone,^39^ adopting a functional test in isolation can lead to unnecessary invasive angiography as a consequence of poor sensitivity and specificity in the detection of CHD. To overcome this, an appropriate strategy would be to combine anatomical and functional tests in order to refine risk stratification and improve selection of patients for revascularization. However, close consideration needs to be given to both the costs and the risks to patients including radiation burden, when multiple testing is incurred.

We would suggest that a safe, cost-effective, and accessible method of achieving combined functional and anatomical assessments is the use of serial exercise electrocardiography testing combined with follow-on CTCA as required. This plays to the strengths of both techniques and protects against their inherent weaknesses. For example, the strength of exercise electrocardiography is the functional assessment of the reproducibility and severity of symptoms combined with high specificity for the presence of obstructive CHD. Its main weakness relates to the poor sensitivity (∼50%) for diagnosing CHD. CTCA compensates for this with a very high sensitivity for CHD and high negative predictive value. The weakness of CTCA in overestimating or poorly defining obstructive disease due to calcification can be mitigated by considering the functional assessment afforded by the exercise ECG. Thus, combining a simple functional test with a highly sensitive anatomical test may represent an ideal strategy in the diagnosis and risk stratification of patients with suspected CHD.

## Future developments

### Combining anatomical and functional imaging

Until recently, an important limitation of CTCA has been the inability to gain functional information about the impact of potential coronary stenoses. However, the development of non-invasive measurements of fractional flow reserve from CTCA (CT-FFR) raises the promise for the potential to gain both anatomical and functional information from a single non-invasive imaging modality. Evidence from recent trials has highlighted that this technique improves the specificity of CTCA and thereby may reduce the number of false-positive tests and unnecessary invasive angiograms.^40,41,42,43^

With the advent of dynamic volume scanners, computed tomography perfusion (CTP) is another promising technique that, when used as an adjunct to CTCA, allows determination of both the anatomical and functional significance of CHD. This technique has yielded sensitivities of 83–91% and specificities of 72–98% when compared with other functional imaging modalities.^44^ Furthermore, it adds incremental diagnostic accuracy to CTCA alone in patients with high calcium scores.^45^ Whilst this technique still has limitations, it has the potential to evolve as an effective addition to CTCA.

### CTCA in the detection of high-risk plaque

**Figure 1:**
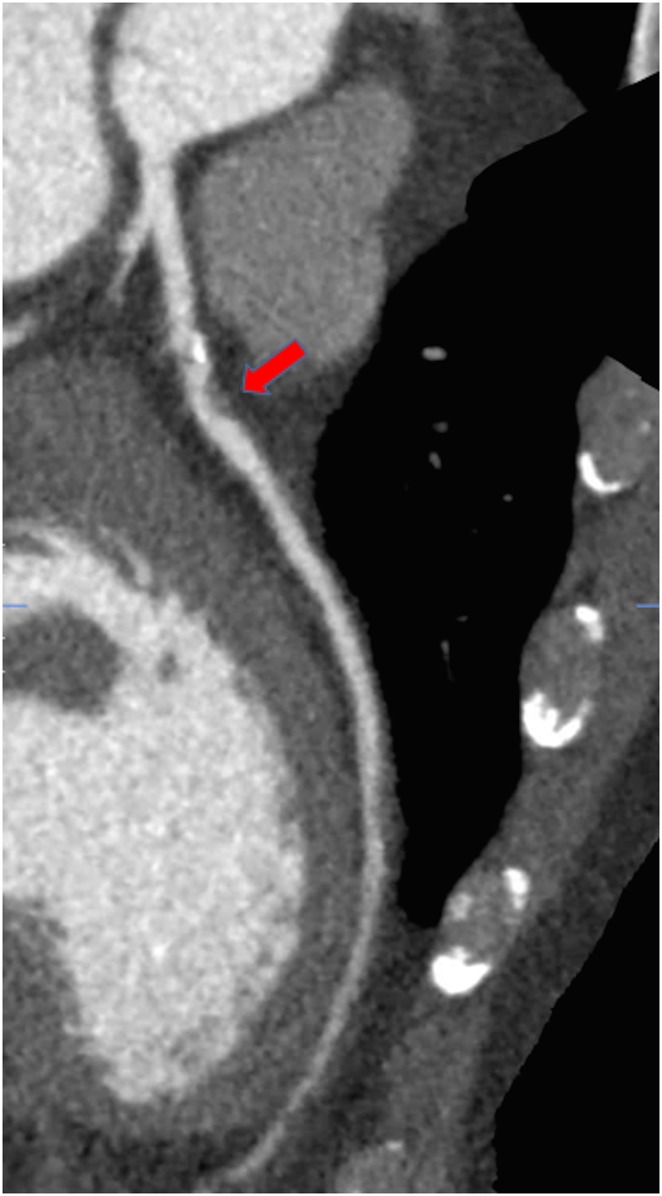
Computed tomography coronary angiography image of a plaque with high-risk characteristics including low attenuation (red arrow).

In addition to identifying anatomically and functionally significant CHD, CTCA has the advantage of being able to detect the presence of features associated with the vulnerable plaque including low attenuation, microcalcification, and positive remodelling.^46^ The presence of high-risk plaque features on CTCA and plaque progression through serial imaging have both been highlighted as independent risk factors for acute coronary syndrome. In a study of over 3000 patients, acute coronary syndrome frequency was 16% in patients with CTCA confirmed high-risk plaque compared with 1.6% of patients with no evidence of high-risk plaque characteristics (*Figure 1*).^46^

**Figure 2:**
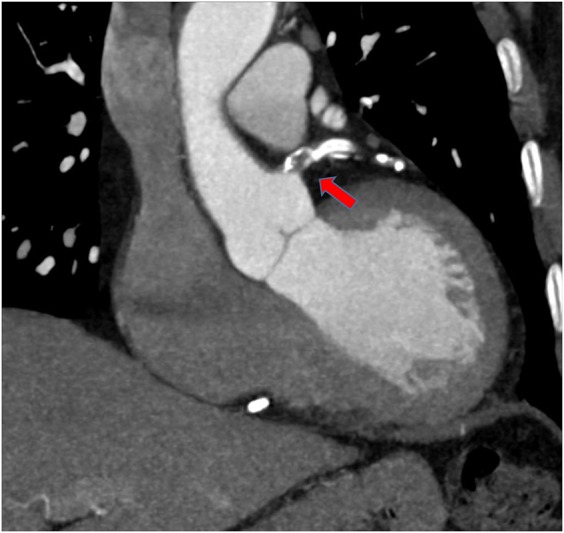
Example of a high-risk proximal left main stem plaque (red arrow) with evidence of positive remodelling and calcification.

The majority of acute ischaemic events arise as a consequence of rupture of a non-flow limiting vulnerable plaque.^47^ Therefore, adopting a functional testing strategy may not identify patients at risk of MI who would benefit from the initiation of treatment. However, despite significant advances in our understanding of the biology of the high-risk plaque,^48^ the clinical utility of documentation of the vulnerable plaque remains uncertain. Future research should focus on the merit of medical or interventional management in patients with non-obstructive high-risk atherosclerotic plaque morphology (*Figure 2*).

## Conclusions

The diagnostic accuracy of CTCA, combined with evidence of its impact on clinical decision-making and outcomes, makes this a powerful and potentially cost-effective tool when integrated into the management of patients with chest pain. In a population of patients with suspected angina secondary to CHD, its use serves to improve patient selection for invasive angiography and revascularization, as well as excluding CHD in those patients who may otherwise be subjected to unnecessary further investigation or life-long medications. Ultimately, CTCA appears to reduce the risk of fatal and non-fatal MI, something that no previous non-invasive diagnostic strategy has been able to achieve.

### Funding

D.E.N. is funded by the British Heart Foundation (CH/09/002) and is supported by a Wellcome Trust Senior Investigator Award (WT103782AIA). He was chief investigator of the SCOT-HEART trial.

**Conflict of interest:** none declared.
